# Sepsis therapies: learning from 30 years of failure of translational research to propose new leads

**DOI:** 10.15252/emmm.201810128

**Published:** 2020-03-16

**Authors:** Jean‐Marc Cavaillon, Mervyn Singer, Tomasz Skirecki

**Affiliations:** ^1^ Experimental Neuropathology Unit Institut Pasteur Paris France; ^2^ Bloomsbury Institute of Intensive Care Medicine University College London London UK; ^3^ Laboratory of Flow Cytometry and Department of Anesthesiology and Intensive Care Medicine Centre of Postgraduate Medical Education Warsaw Poland

**Keywords:** animal models, cytokine storm, personalized medicine, reprogramming, sepsis, Immunology, Microbiology, Virology & Host Pathogen Interaction, Molecular Biology of Disease

## Abstract

Sepsis has been identified by the World Health Organization (WHO) as a global health priority. There has been a tremendous effort to decipher underlying mechanisms responsible for organ failure and death, and to develop new treatments. Despite saving thousands of animals over the last three decades in multiple preclinical studies, no new effective drug has emerged that has clearly improved patient outcomes. In the present review, we analyze the reasons for this failure, focusing on the inclusion of inappropriate patients and the use of irrelevant animal models. We advocate against repeating the same mistakes and propose changes to the research paradigm. We discuss the long‐term consequences of surviving sepsis and, finally, list some putative approaches—both old and new—that could help save lives and improve survivorship.

## Graphical abstract

This review recapitulates our knowledge on sepsis and its long‐term consequences, the lack of therapeutic advances in the last decades, and proposes new approaches to improve sepsis survival.

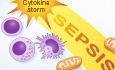

**Glossary**
Autophagy: A natural, regulated mechanism of the cells that removes unnecessary or dysfunctional components within the cytoplasm. It allows the orderly degradation and recycling of cellular components.
Cecal Ligation and Puncture (CLP): A common rodent model of sepsis following surgery with ligation and puncture of the cecum, which induces a polymicrobial septic insult (peritonitis).
Compartmentalization of the immune response: A differential polarization of the immune response in different tissues and organs that is regulated by the local microenvironments ending to specific responses of cells.
Endothelial permeability: An inflammation‐induced impairment of the dynamic barrier between endothelial cells embedding vessels that leads to the leak of small plasma molecules.
Endotoxin tolerance: A state induced mainly in monocytes/macrophages by endotoxin or by exogenous or endogenous inflammatory stimuli. Cells become refractory to secondary challenge with endotoxin or other inflammatory stimuli. The phenomenon is regulated at multiple levels (epigenic, miRNA, negative signaling, cross‐talk with immunosuppressive cells…).
Endotype: A subtype of a clinical syndrome that shares common pathophysiological mechanisms.
Hibernation: A metabolic‐bioenergetic shutdown allowing the organs to retain the capacity to recover after the insult has passed.
Immunosenescence: A natural decline of the immune system functions with aging.
Microbiome: A collection of microorganisms that can be found in or on multicellular organism in the context of anatomical location (gut microbiome, skin microbiome, lung microbiome).
Neutrophil Extracellular Traps (NETs): DNA structures actively released during programmed death of neutrophils, which primary aim to entrap and kill the pathogens. A dysregulated production of NETs can be detrimental to the host and induce pathologies.
Pathobiome: A disease‐related changed in microbiome.
Persistent inflammation, immunosuppression, and catabolism syndrome (PICS): A phenotype underlying the chronic critical illness, initiated early in the course of disease. This is perpetuated by the release of damage‐associated molecular patterns (DAMPs) associated with signs of immunosuppression and altered metabolism.
Reprogramming: The modified responsiveness of innate immune cells in response to a second stimulus. Depending on the nature of the first challenge, it can be similar to endotoxin tolerance or to priming (also referred to as innate immune memory).
Sepsis: A life‐threatening organ dysfunction caused by a dysregulated host response to infection.
Septic shock: A subset of sepsis in which particularly profound circulatory, cellular, and metabolic abnormalities are associated with a greater risk of mortality than with sepsis alone.
Sequential Organ Failure Assessment Score (SOFA): A simple tool to assess the degree of organ dysfunction, to track disease progression, and to predict outcome; it comprises six categories covering abnormalities in respiratory, cardiovascular, hepatic, coagulation, renal, and neurological systems.
Sick euthyroid syndrome: A condition in which low levels of thyroid hormones are found in patients with non‐thyroid illness.
Theranostics: An individualized medicine approach combining targeted therapeutics and diagnostic test
Translational research: Preclinical research aiming at understanding the physiopathology and the molecular mechanisms of a human disease or at proposing new therapeutic approaches to improve health outcome. Laboratory animals are often used to mimic human diseases.

## Sepsis: a new WHO global health priority

**Figure 1:**
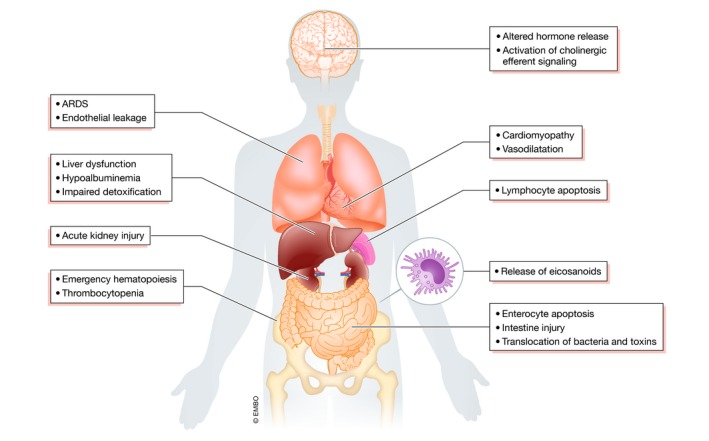
Summary of sepsis pathophysiology Upon direct activation of immune and endothelial cells by the pathogen‐associated molecular patterns, there is a massive release of inflammatory mediators which affect each body system. Inflammatory response activates the central nervous system, which acts by cholinergic anti‐inflammatory impulsion and altered neuroendocrine response to control the body response to infection and increase chances of survival. Cardiovascular dysfunction plays a central role in the pathogenesis of sepsis with the major role of vasoplegia, hypovolemia, microcirculation perturbations, and cardiomyopathy. Altered endothelium and inflammatory cells lead to the development of acute respiratory distress syndrome (ARDS). The direct action of cytokines and toxins, together with decreased blood flow, leads to acute kidney injury (AKI). Inflammatory response and ischemia alter gut permeability which enables entry of bacteria and their metabolites into the tissues. Both bacterial products and inflammatory mediators affect bone marrow progenitor cells enhancing the emergency myelopoiesis. Most often, the failure of multiple organs is present, which has significant consequences as there is a cross‐talk between injured organs which further perpetuates their dysfunction. For a more detailed perspective on organ failure in sepsis, we refer to a recent review (Lelubre & Vincent, 2018).

Sepsis was previously considered an infectious systemic inflammatory response syndrome (SIRS; Bone *et al*, 1992; Levy *et al*, 2003). However, recognizing that this host response is usually part and parcel of an individual's appropriate defense against infection, sepsis was re‐defined in 2016 (“Sepsis‐3”) as “*a life‐threatening organ dysfunction caused by a dysregulated host response to infection*” (Fig 1) (Singer *et al*, 2016). For clinical operationalization, organ dysfunction is identified by an increase from baseline of ≥ 2 points in the Sequential Organ Failure Assessment (SOFA) score. Patients are identified as having septic shock when vasopressors are required to maintain a mean arterial pressure ≥ 65 mmHg and when serum lactate levels remain > 2 mmol/l (>18 mg/dl) despite adequate volume replacement.

In 2017, the WHO passed a resolution, recognizing sepsis as a global health priority (Reinhart *et al*, 2017). A recent investigation estimated that in 2017, sepsis occurred in 48.9 million people worldwide, ending to the death of 11 million patients (Rudd *et al*, 2020). These estimates are more than double previous global figures (Fleischmann *et al*, 2016). This increase is probably attributable to inclusion of more data from low‐income and middle‐income countries, locations where sepsis incidence and mortality are considerably higher and for which data were previously under‐represented. It is also unclear how many people die “*of”* or “*with”* sepsis as the majority of deaths, at least in developed countries, occur in patients who are elderly, frail, and/or have significant underlying comorbidities. National Health Service data from England suggest that 77.5% of deaths occur in those aged ≥ 75 years (Singer *et al*, 2019). The incidence of sepsis is also reported to be rising at an alarming rate though such data should be treated cautiously. While in part due to an aging population and increasing invasive medical interventions, increased awareness of the condition and significant financial incentivizations to use administrative sepsis codes play an important part in the reported incidence increase. Indeed, Rhee *et al* (2017) indicated that sepsis incidence based on Sepsis‐3 clinical criteria was stable over a 5‐year period in 409 US hospitals (0.6% relative increase per year), yet had risen by 10.3% per year according to insurance claims data. By 2014, the incidence of sepsis was twice as high, affecting 12% of the total hospital cohort, using claims‐based data. This large rise in denominator also generated a spurious reduction in the rate of death or discharge to hospice, namely 4.5% per year using claims data compared to 1.3% using clinical data.

These conflicting data on incidence and outcomes reflect considerable inconsistencies within the literature, particularly as most of the data are extracted from hospital administrative databases or insurance claims data that are prone to the confounders described above. Nonetheless, it is fair to say that sepsis continues to be a significant healthcare issue with high mortality (approximately 25% for sepsis and 40–50% for septic shock (Shankar‐Hari *et al*, 2017) and morbidity, and with a major impact on resource utilization. This continues after discharge from hospital, as survivors often suffer post‐sepsis symptoms such as fatigue, neuromuscular weakness, chronic pain, post‐traumatic stress disorder, cognitive impairments, and depression. Furthermore, sepsis has a high price to insurance systems (Arefian *et al*, 2017).

Notwithstanding coding anomalies, mortality from sepsis does appear to be improving. This is mainly the consequence of improved health care with earlier recognition and treatment, and less iatrogenic harm. Multiple studies in intensive care patients have demonstrated an injurious impact from over‐exuberant use of mechanical ventilation, fluid therapy, sedation, nutrition, and inotropes, to name but some interventions. Unfortunately, these outcome improvements cannot be attributed to introduction of new therapies, nor to a better understanding of the pathophysiology. This is not for the lack of clinical trials undertaken over the last 30 years, started after successful outcomes in animal models that are often reported in high impact journals. The failure of this translational research should raise questions within the medical and scientific communities, and inspire them to avoid perpetuating expensive experimental animal studies and clinical trials without modifying the underlying paradigms. Several published articles have already explored the reasons for these failures (Rittirsch *et al*, 2007; Dyson & Singer, 2009); however, no significant changes have emerged.

## Well‐established processes and new concepts

### Molecular players

**Figure 2:**
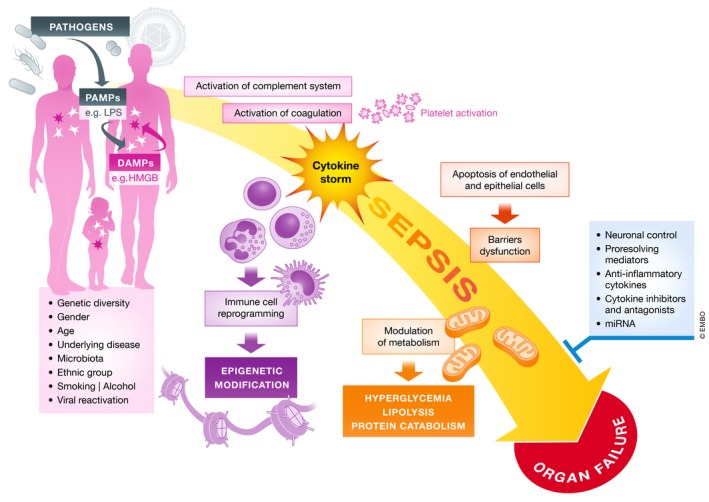
Summary of the players and pathophysiological events occurring and influencing sepsis Complex interactions between genetic and chronic health status determine the host response to pathogens. The magnitude and variety of humoral and cellular response may lead to organ dysfunctions, which are a key denominator of sepsis in comparison with other forms of infection.

New findings are constantly enlightening our understanding of the pathophysiological processes involved in sepsis. Most of the players involved have likely been identified (Fig 2). The host response is initiated by both pathogen‐associated molecular patterns (PAMPs), such as endotoxin and lipoteichoic acid, and damage‐associated molecular patterns (DAMPs), such as heat shock proteins, high mobility group box 1, nucleotides, and mitochondria released from injured host cells.

Activation of the complement system has long been recognized as a potentiator of the inflammatory response during sepsis, in particular the role played by anaphylatoxin C5a (Riedemann *et al*, 2002). An inhibitor of C5 cleavage showed reduced organ damage and better outcome in a baboon model of *Escherichia coli* sepsis (Keshari *et al*, 2017). Similarly, modifying the coagulation cascade has been advanced with numerous successful approaches in animal sepsis models targeting pro‐coagulant factors (Levi & van der Poll, 2017).

Various other molecular players have been identified as performing a beneficial role in murine cecal ligature and puncture (CLP) models of sepsis. These include IL‐33 and IL‐38 (Li *et al*, 2016; Xu *et al*, 2018), pro‐resolving mediators such as resolvin D2 (Spite *et al*, 2009), and the cell surface nucleotide‐metabolizing enzyme CD39 (Csóka *et al*, 2015). Acetylcholine produced by neurons and by a specific subset of T‐lymphocytes in response to norepinephrine is considered a significant component in the neuronal control of inflammation (Rosas‐Ballina *et al*, 2011), though the role of the vagus nerve in neuro‐immune cross‐talk is controversial (Martelli *et al*, 2014; Pavlov *et al*, 2018). In a case report of successful use of experimental electroacupuncture to protect against sepsis, dopamine was shown to be the primary beneficial mediator (Torres‐Rosas *et al*, 2014).

### Circulating cells

**Figure 3:**
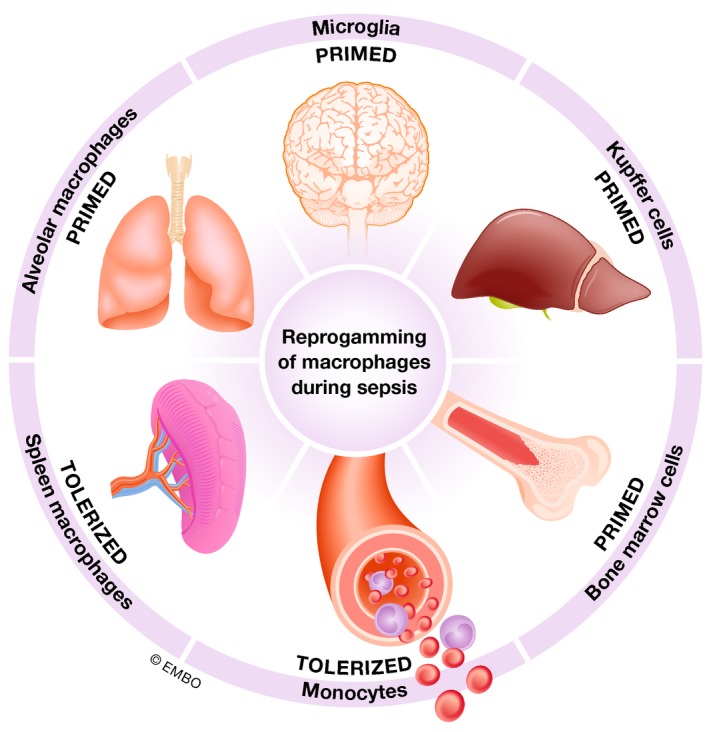
Compartment‐specific reprogramming of the macrophages in sepsis Microenvironment modulates the response of macrophages during sepsis. Therefore, the features of cells from one compartment cannot be generalized upon others.

Sepsis is associated with the reprogramming of circulating leukocytes (Cavaillon *et al*, 2005). The term “immunosuppression” is widely used to qualify this phenomenon, which is inappropriate and misleading (Cavaillon & Giamarellos‐Bourboulis, 2019). The *ex vivo* behavior of leukocytes is greatly influenced by the compartment they are derived from (Rasid & Cavaillon, 2018; Fig 3). Blood leukocytes display reduced capacities to proliferate and to produce cytokines and antibodies. Monocytes show reduced expression of HLA‐DR mRNA, while neutrophils show an increased expression of CD64 mRNA. Notably, recruited monocytes within the lungs express 3.5‐fold more membrane HLA‐DR compared to circulating monocytes (Skirecki *et al*, 2016). Lymphocytes exhibit enhanced spontaneous apoptosis while neutrophils’ anti‐CD24‐induced apoptosis is reduced (Parlato *et al*, 2014). These alterations occur rapidly and in proportion to the intensity of the insult, be it sepsis or other critical illnesses such as trauma, hemorrhagic shock, major surgery, resuscitation after cardiac arrest, and pancreatitis (Kim *et al*, 2010; Timmermans *et al*, 2016). As expected, the altered capacity of circulating monocytes is associated with an enhanced expression of inhibitory signaling molecules (Escoll *et al*, 2003; Adib‐Conquy *et al*, 2006), histone modifications (Bomsztyk *et al*, 2015)**,** and specific miRNAs (Zhou *et al*, 2015; Reithmair *et al*, 2017). Some miRNAs can attenuate sepsis‐associated alterations in myeloid cells, endothelial cells, and in the myocardium (Zhou *et al*, 2017; Sisti *et al*, 2018). Circulating extracellular vesicles containing miRNAs may be a novel mechanism of intercellular communication during sepsis (Real *et al*, 2018). Whether these extracellular vesicles have an inhibitory role via delivery of miRNAs or display a pro‐inflammatory potential via their capacity to activate monocytes (Danesh *et al*, 2018) remains to be elucidated.

In addition to their role in blood clotting, platelets play an important role in multiple biological functions. This diversity relates to their capacity to sense and respond to infectious agents and to release significant amounts of inflammatory mediators (Garraud *et al*, 2013). Platelets can bind to numerous circulating leukocytes, particularly neutrophils (Gawaz *et al*, 1995), favoring the formation of neutrophil extracellular traps (Clark & Coopersmith, 2007), neutrophil recruitment LPS‐induced lung injury (Grommes *et al*, 2012), and neutrophil rolling onto the endothelium and margination outside venules (Sreeramkumar *et al*, 2014). Thrombocytopenia is a hallmark of sepsis and may relate to decreased production and/or increased destruction. In a recent study, 38% of sepsis patients had low or very low platelet counts; such patients had a higher clinical illness severity score and an increased mortality (Claushuis *et al*, 2016). Of note, levels of circulating RANTES (CCL5), a chemokine mainly derived from platelets, were lower in the most severely ill patients with poor outcomes (Cavaillon *et al*, 2003).

It should be stressed that for most molecular and cellular players, conflicting contributions have been revealed. While platelets contribute to inflammation, they have also been shown to decrease organ damage in LPS‐treated mice (Xiang *et al*, 2013) through a C‐type‐lectin‐like‐2 (CLEC‐2)‐dependent process (Rayes *et al*, 2017).

### Endothelium

The pivotal role of endothelial cells in the pathogenesis of sepsis is well recognized. Most endothelium physiological functions are disturbed, leading to increased vascular permeability, activation of coagulation, and participation in the inflammatory response (Opal & van der Poll, 2015). Some recently reported aspects of endothelial injury in sepsis may also contribute significantly to mortality (Johansen *et al*, 2015). The role in sepsis of the angiotensin‐1,‐2/Tie2 pathway, an important regulatory axis of the endothelium, was recently reviewed (Leligdowicz *et al*, 2018). High levels of angiopoietin‐2 in pneumonia patients contributed to endothelial permeability and were related to poor outcomes, while angiopoietin‐1 was shown to ameliorate vascular leak (Gutbier *et al*, 2018). Mechanistically, a reduction of the histone deacetylase, sirtuin 3, leads to the increase in angiopoietin‐2 and fall in angiotensin‐1. These changes also contribute to the loss of pericytes (Zeng *et al*, 2016). Furthermore, the Tie‐2 receptor is downregulated in sepsis at both transcriptional and protein levels (Thamm *et al*, 2018). Another mediator in sepsis‐induced endothelial dysfunction is microRNA‐155, which promotes bioenergetic deterioration, contractile dysfunction, pro‐inflammatory activation, and downregulation of the angiotensin type 1 receptor (Vasques‐Novoa *et al*, 2018). A novel insight into the mechanism of endothelial cell death in sepsis described an intracellular endotoxin‐sensing machinery in the induction of endothelial cell pyroptosis (Cheng *et al*, 2017).

### Epithelium

Maintenance of the gut barrier can be severely impaired in sepsis by enterocyte loss from apoptosis and pyroptosis (Mandal *et al*, 2018), and a rapid decline of tight junctions following reduced expression of claudins and occludin (Yoseph *et al*, 2016). The observations of enterocyte injury and increased gut wall permeability led to the concept of the “leaky gut” as the motor of multi‐organ dysfunction in critical illness, acting as a source of bacteria and bacterial toxins (Carrico *et al*, 1986). Although this hypothesis has not gained sufficient traction in clinical studies, and patients do not seem to die from bacteremia (Savage *et al*, 2016), updated and modified insights offer attractive new pathophysiological concepts, in particular the protective role of the microbiome (Alverdy & Krezalek, 2017; Meng *et al*, 2017) and its modulation by probiotics (Angurana *et al*, 2018). However, dissemination of intestinal bacteria does not seem to be an essential event as gut‐derived DAMPs can travel through the lymph and cause distant organ injury (Reino *et al*, 2011).

### Endocrinopathy

The central nervous system is stimulated during sepsis by efferent impulsion, inflammatory cytokines, and PAMPs that can enter the blood‐brain barrier at specific locations (Annane, 2016). These signals modify the central endocrine axes that orchestrate metabolic and immune responses, and these change over time. For a detailed description of endocrine alterations in sepsis, we recommend a recent review by Ingels *et al* (2018). Briefly, early endocrine responses to a critical insult are generally protective. However, the degree of activation of the hypothalamic–pituitary–adrenal (HPA) axis represents a significant stress response that correlates with poor outcomes (Vassiliadi *et al*, 2014). Release of adrenocorticotropic hormone is increased, while the high serum concentration of cortisol is also due to impaired clearance (Annane, 2016). The potentially advantageous effects of early high cortisol production are counteracted by peripheral glucocorticoid resistance mediated by pro‐inflammatory cytokines. However, a subgroup of patients could not mount an efficient cortisol response due to inhibition of the HPA axis and would potentially benefit from glucocorticoid supplementation (Annane, 2016).

Plasma catecholamine levels are also elevated, more so in eventual non‐survivors (Boldt *et al*, 1995), with multiple negative consequences including impaired myocardial function, inhibition of both innate and adaptive immunity yet enhanced pathogen virulence and growth, pro‐coagulopathic effects, altered gut motility, lipolysis, and insulin resistance (Andreis & Singer, 2016).

Sepsis also affects thyroid function by an early increase in thyrotropin (TSH) production but a reduction in both thyroxine and the more active triiodothyronine (Peeters *et al*, 2006). This condition, often called the sick euthyroid syndrome or the low T3 syndrome, also correlates with a poor outcome (Angelousi *et al*, 2011). A trial of thyroxine treatment in critically ill patients however resulted in higher mortality (Acker *et al*, 2000).

Another pituitary hormone, growth hormone (GH), is initially upregulated in septic patients and correlates with disease severity (Schuetz *et al*, 2009). However, its downstream effectors are decreased in the serum (Baxter *et al*, 1998). High levels of GH stimulate lipolysis and antagonize insulin. Yet, two multi‐centre studies of growth hormone treatment in critically ill patients showed a doubling in mortality rates (Takala *et al*, 1999).

Apart from disturbances in the central hormonal axes, there is a growing interest in the role of intestine‐released hormones and fat‐derived adipokines in the pathogenesis of sepsis. However, it remains unclear whether altered levels of these hormones and adipokines contribute to pathology, or are simply epiphenomenal and reflective of dysregulated homeostasis. Examples include insulin, the incretins (GLP‐1 and GIP), ghrelin, and leptin. High and persisting levels of glucagon‐like peptide‐1 in septic patients are associated with an increase in mortality and functional disability (Brakenridge *et al*, 2019). Ghrelin, involved in appetite stimulation, increases in the plasma of septic patients (Nikitopoulou *et al*, 2019), and active ghrelin levels are inversely correlated with the SOFA organ dysfunction score and length of ICU stay. In short‐term preclinical models of sepsis, ghrelin has been shown to decrease pro‐inflammatory cytokine levels, stimulate proliferation of T cells, and attenuate the decrease in serum levels of IGF‐1 (Faim *et al*, 2019); however, we found that ghrelin infusion in a long‐term rodent fecal peritonitis model had no impact on outcomes (Hill *et al*, 2017). Plasma insulin levels are variably reported as raised or depressed in sepsis, but insulin resistance is commonplace regardless, with subsequent hyperglycemia‐inducing toxicity. The optimal degree of glycemic control nonetheless remains controversial (Gunst *et al*, 2019). Peripheral resistance indicates abnormalities at, or distal to, the insulin receptor (Van Bogaert *et al*, 2011). Similar issues are likely to exist for other hormonal pathways, such as glucocorticoids and growth hormone.

### Metabolism

The importance of alterations in metabolism during sepsis is well recognized and is part of the current definition of septic shock (Singer *et al*, 2016). With peripheral insulin resistance and increased lipolysis and proteolysis, there is a general shift toward fat and protein utilization. This in turn impacts upon immune functionality (Balmer & Hess, 2017) and metabolic efficiency. However, many questions remain unanswered (Van Wyngene *et al*, 2018). Many clinical and preclinical data indicate sepsis‐induced mitochondrial dysfunction in multiple tissues (Brealey *et al*, 2002; Singer, 2014) with a shift toward anaerobic (glycolytic) metabolism. While anaerobic metabolism can boost the antimicrobial capacities of immune cells, it may also contribute to their loss of function (Balmer & Hess, 2017). In the course of sepsis, monocytes develop broad defects in both glycolysis and oxidative phosphorylation that are related to the phenomenon of endotoxin tolerance and which can be restored by interferon‐gamma (IFNγ) treatment (Cheng *et al*, 2016). A recent genome‐wide array study (Davenport *et al*, 2016) proposed two signatures of gene expression patterns, with one related to higher mortality and features of immunosuppression. This subgroup showed upregulated expression of hypoxia‐inducible factor 1 alpha (HIF1α), HIF2α, and lactate dehydrogenase A, but downregulation of the mechanistic target of rapamycin (mTOR).

As failed organs show minimal to no evidence of cell death, a global metabolic shutdown is postulated as the primary mechanism of organ dysfunction in sepsis (Singer, 2014). This state, akin to hibernation, may even represent an adaptive mechanism, allowing restoration of organ function once the inflammatory process resolves (Singer *et al*, 2004; Stanzani *et al*, 2020). Notably, sepsis non‐survivors are characterized by subnormal levels of muscle ATP (Brealey *et al*, 2002), while maintenance of hepatic gluconeogenesis was shown to be crucial in establishing disease tolerance (Weis *et al*, 2017). These data suggest that adaptive pathways can spill over into maladaptation if sufficiently dysregulated.

Impaired bioenergetics under septic conditions have also been shown to affect energy‐consuming functions in multiple types of epithelial cells. For example, pneumocytes downregulate the Na^+^,K^+^‐ATPase pump, which is responsible for fluid clearance in alveoli (Vadasz *et al*, 2008), while renal tubular epithelial cells decrease sodium channel expression (Gomez *et al*, 2015). Controlled autophagy may be an important process required for epithelial cell survival under septic conditions (Oami *et al*, 2018).

### Microbiome/Pathobiome

A growing body of evidence demonstrates that gut microbiota interacts with the gut epithelium and the host's immune system. The microbiome and its metabolites are essential regulators of health under steady‐state conditions. A severe insult such as infection or trauma rapidly induces profound changes in the intestinal ecosystem (dysbiosis), leading to loss of diversity of the physiological, mostly anaerobic flora (Ojima *et al*, 2016), unification of the microbiome composition at different sites (Rogers *et al*, 2016), and outgrowth of pathological species that are often nosocomial (Alverdy & Krezalek, 2017). The microbiome is compromised further by concurrent use of broad‐spectrum antibiotics, even with only a few doses (Iapichino *et al*, 2008; Ferrer *et al*, 2017).

The gut is not the only site harboring microbiota. For example, the skin and lung are also colonized with their respective microbiome, but their biology is less well understood (Gilbert *et al*, 2018). Recently, both intestine and upper airways microbiota were shown to regulate lung immunity (Brown *et al*, 2017). While the role of the microbiome in other tissues such as the kidney remains to be specified in the pathophysiology of sepsis, the skin microbiome has recently been shown to favor the pathogenicity of certain bacteria such as *Staphylococcus aureus* (Boldock *et al*, 2018).

Notwithstanding iatrogenic administration of antibiotics and gastric acid suppressants, the host's overall changes during critical illness promote the emergence of the pathobiome (disease‐related flora; Zaborin *et al*, 2014; Gilbert *et al*, 2016). This includes supranormal levels of catecholamines that induce pathogen growth, virulence, and biofilm formation, as well as directly impacting on the microbiome (Dickson *et al*, 2015; Sarkodie *et al*, 2019).

Numerous studies suggest that the relationship between the host and microbiome/pathobiome is bidirectional (Kelly *et al*, 2015; Sarkodie *et al*, 2019). Of note, gut‐specific bacteria can dominate the lung microbiome in septic patients, and this change correlates with alveolar TNF expression (Dickson *et al*, 2016). Preserving the physiological gut microbiome could protect the host from pneumonia (Schuijt *et al*, 2016), sepsis (Wilmore *et al*, 2018), or melioidosis (Lankelma *et al*, 2017) by maintaining the gut barrier (Fox *et al*, 2012), enhancing immunity, and protecting the brain (Li *et al*, 2018). Clinical data have indirectly shown the impact of the disturbed microbiome on susceptibility to sepsis (Prescott et al, 2015a; Baggs *et al*, 2018). Colonization with *Enterococci* at ICU admission was related to a higher risk of subsequent infection and mortality (Freedberg *et al*, 2018). The abovementioned findings shine a new light on the gut and the microbiome/pathobiome as significant players in the pathophysiology of sepsis. The development and decreasing cost of metagenomics and metabolomics will likely move this field rapidly. Recent murine trials showed the potential of modulating the microbiome with a high fiber diet (Morowitz *et al*, 2017), while the first successful fecal microbiota transplantation used as a salvage therapy in critically ill ICU patients has also been reported (Wei *et al*, 2016).

## Short‐ and long‐term consequences of surviving sepsis

Surviving sepsis creates a plethora of new healthcare challenges. Up to 40% of patients who survive the early phase of sepsis develop chronic critical illness, a state defined as a prolonged (> 14 days) ICU stay with sustained organ dysfunction (Stortz *et al*, 2018). Many of these patients do not leave hospital alive or are not fit enough to return to their own homes. In the longer term, there is a higher mortality risk, poorer health status, and an increased requirement for more medical interventions and rehospitalizations (DeMerle *et al*, 2017). This so‐called post‐intensive care syndrome reflects new onset (or worsening of preexisting) neurocognitive deterioration, psychiatric complications, and/or physical impairments (Needham *et al*, 2012).

### Neuropathy

Acute brain dysfunction is a frequent and severe early sign of sepsis present in about 50% of cases admitted to the ICU (Sonneville *et al*, 2017). It can manifest as confusion, delirium, drowsiness, coma, seizures, or with focal neurological signs. Sepsis‐associated encephalopathy (SAE) includes not only early brain dysfunction but also the longer‐term impairments seen in sepsis survivors. Brain lesions can be found in approximately a third of patients with clinically diagnosed SAE (Orhun *et al*, 2018). Two distinct patterns of neuroaxonal injury—ischemic and diffuse—were identified using magnetic resonance imaging and immunohistopathologic analysis of post‐mortem brains (Ehler *et al*, 2017). The pathophysiology of SAE is incompletely understood, but is driven by systemic inflammation, which leads to disruption of the blood–brain barrier and ingress of bacterial toxins, pro‐inflammatory monocytes, and neutrophils into the brain tissue (Kuperberg & Wadgaonkar, 2017; Tauber *et al*, 2017). Early infiltration of NK cells enhances monocyte recruitment (He *et al*, 2016). Pro‐inflammatory cytokines and toxins activate microglia and astrocytes (Hasegawa‐Ishii *et al*, 2016), and fueling local inflammation (Zrzavy *et al*, 2018). Together with afferent impulsion, the inflamed microenvironment induces neuronal changes. At the functional level, SAE is related to decreased brain oxygenation (Wood *et al*, 2016), metabolic shifts (Hara *et al*, 2015), and excitotoxicity (Mazeraud *et al*, 2016). The aforementioned mechanisms may be sustained in sepsis survivors with long‐term consequences such as cognitive and functional impairment (Annane & Sharshar, 2015), depression (Davydow *et al*, 2013), stress disorders (Wintermann *et al*, 2015), and neurological complications (Reznik *et al*, 2017). Recent animal studies support the causative link between sepsis and long‐term psychological dysfunction (Barichello *et al*, 2019). Other improvements of cognitive impairment after sepsis have been achieved with some of the treatments known to protect mice against sepsis‐induced death, such as anti‐HMGB‐1 antibodies (Chavan *et al*, 2012; Gentile & Moldawer, 2014; Stevens *et al*, 2017) or electroacupuncture (Han *et al*, 2018). Peripheral nerves can also be affected by sepsis inducing a critical illness polyneuropathy. Loss of axonal fibers affecting motor and/or sensory nerves can be observed in skin biopsies, with impairment of signal conduction (Hermans & Van den Berghe, 2015; Axer *et al*, 2016).

### Myopathy

Similar mechanisms to neuropathy can also induce sepsis‐related myopathy. Either one and/or both conditions (collectively known as ICU‐acquired weakness) can worsen the patient's outcome, lead to prolonged mechanical ventilation (Sharshar *et al*, 2009), and carry long‐term consequences (Hermans & Van den Berghe, 2015). Sepsis‐related myopathy is characterized by the loss of muscle mass, a fall in force‐generating capacity, and altered bioenergetics (Hermans & Van den Berghe, 2015). Critically ill patients face multiple risk factors causing myopathies such as immobilization, local inflammation‐induced by systemic mediators, and nutritional defects (Friedrich *et al*, 2015). Mechanisms responsible for septic myopathy include sodium channel dysfunction with upregulation of non‐selective channels (Balboa *et al*, 2018), ubiquitin–proteasome pathway protein proteolysis, proteasome activation and increased autophagy (Wollersheim *et al*, 2014; Preau *et al*, 2019), changes in intracellular calcium levels leading to excitation‐contraction uncoupling (Batt *et al*, 2013), and mitochondrial derangements with an increase in free radical generation (Zolfaghari *et al*, 2015). Although autophagy is among the mechanisms of protein loss, its balanced activation can protect muscles from the accumulation of toxic proteins (Morel *et al*, 2017). Through activation of the TNF and mTORC1 pathways, sepsis impairs the physiological anabolic response of muscles to contraction, thereby slowing down recovery from atrophy (Steiner & Lang, 2015). Recovery from sepsis is related to increased muscle protein synthesis and decreased autophagy, but in juxtaposition, proteasome activity is upregulated, possibly hindering myosin restoration (Crowell *et al*, 2017). Interestingly, the satellite cells, which serve as muscle progenitor cells, are severely impaired by sepsis and are not able to regenerate injured muscle (Rocheteau *et al*, 2015) due to upregulated oxidative phosphorylation and loss of mitochondrial mass. In consequence, these alterations lead to the loss of satellite cells.

The incidence of ICU‐acquired weakness negatively affects the quality of life of survivors (Hermans & Van den Berghe, 2015), and recovery from ICU‐acquired weakness can take up to one year after discharge, or even be permanent, especially with neuropathy.

### Altered immunity

Sepsis survivors are at higher risk of recurrent infection, and this is a major reason for re‐hospitalization (Prescott *et al*, 2015b). Whether recurrent infections contribute substantially to long‐term mortality is unclear, but they worsen quality of life and constitute an additional risk to survivors (Shankar‐Hari *et al*, 2016). Susceptibility to infection suggests sustained immune impairments in sepsis survivors, which are only now being identified. Currently recognized mechanisms of long‐term immune impairment include expansion of myeloid‐derived suppressor cells (Mathias *et al*, 2017), a high proportion of regulatory T cells (Cavassani *et al*, 2010), disturbed T‐cell recovery (Condotta *et al*, 2013), and multiple epigenetic modifications within various cell types (Hassan *et al*, 2018). A recent study also revealed persistently increased inflammatory mediators such as HMGB1, TNF, IL‐7, and resolvins, even at 1 year after hospital discharge (Riche *et al*, 2018).

**Figure 4:**
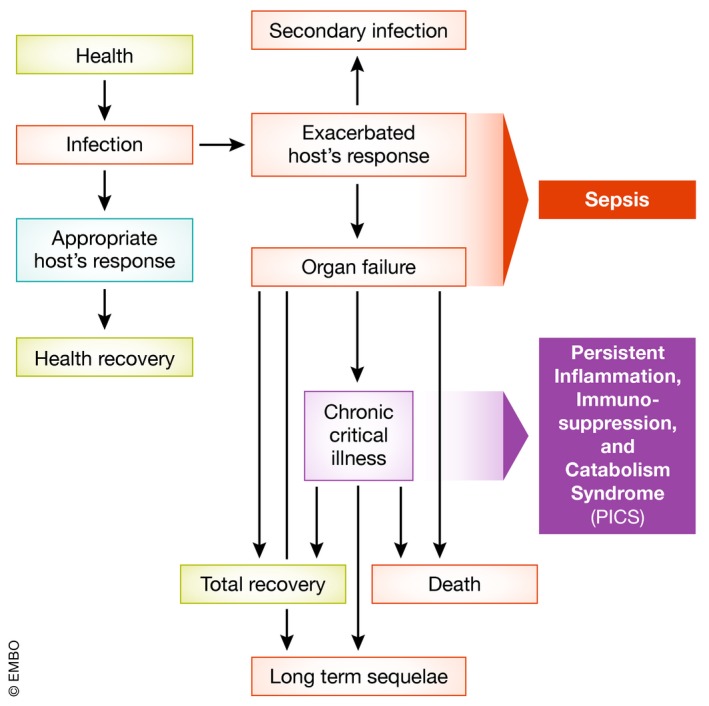
Long‐term sequel of sepsis Most of the sepsis cases occur in patients with chronic comorbidities. Within days to weeks, some patients can be healed while some will succumb due to acute organ dysfunctions. However, a high frequency of patients develop chronic critical illness (ICU stay for more than 14 days). This condition is caused mechanistically by the persistent inflammation, immunosuppression, and catabolism syndrome (PICS). During this phase, some patients die due to organ dysfunctions, and some develop secondary infections. A group of chronic critical illness patients still can fully recover. However, most of the patients will experience the worsening of their chronic conditions and suffer from the onset of new ones.

These alterations in immune status do not necessarily reflect a global defect (Rubio *et al*, 2019). For example, sepsis could improve tumor‐specific CD8^+^ T‐cell responses (Danahy *et al*, 2019). TCR‐dependent T‐cell function is somewhat augmented, pointing against a paradigm of generalized immunosuppression in sepsis survivors (Borken *et al*, 2017). However, there is an incomplete recovery of the TCR reservoir after sepsis, and this process is in part dependent on the gut microbiome (Cabrera‐Perez *et al*, 2016). Importantly, these changes are accompanied by a smoldering low‐grade inflammation that induces a catabolic shift and may perpetuate disturbed myelopoiesis (Horiguchi *et al*, 2018). This phenotype has been coined “persistent inflammation, immunosuppression, and catabolism syndrome” (PICS) and can be viewed as a mechanism underlying chronic critical illness (Gentile *et al*, 2012; Horiguchi *et al*, 2018; Fig 4).

## What went wrong? Explaining 30 years of failure of translational research

### Inappropriate animal models

For the last thirty years, using genetically engineered mice or animals treated with antibodies or drugs that target a given molecule, investigators have successfully demonstrated a deleterious contribution from a large number of molecular players and pathways during sepsis, and beneficial consequences following their neutralization. However, none have resulted in positive clinical trials. This may relate to the limited predictive value of preclinical models of disease (Plenge *et al*, 2013).

**Figure 5:**
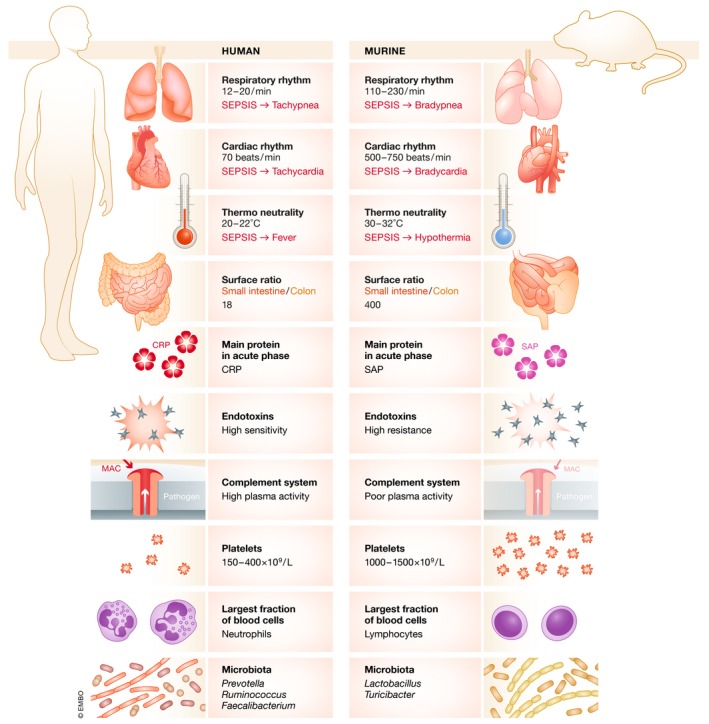
Some keys differences in murine and human physiology that affect the response to sepsis (CRP —C‐reactive protein, MAC—membrane attack complex, SAP—serum amyloid protein)

It is indeed essential to acknowledge significant differences that exist between mice and humans (Fig 5, Table 1), not only in terms of physiology but also with regard to the response to a septic insult. For instance, as opposed to humans, mice develop bradypnea rather than tachypnea, and bradycardia rather than tachycardia (Iskander *et al*, 2013; Hoover *et al*, 2015). When maintained at room temperature, septic mice are under cold temperature stress (Karp, 2012) and usually display hypothermia in line with illness severity (Zolfaghari *et al*, 2013). If their temperature is kept close to thermoneutrality, mice can generate a fever, and their ability to manage sepsis significantly improves (Jiang *et al*, 2000). The different circadian rhythm between mice and humans also impacts upon the immune response and the timing of expression of key regulators such as HIF1α (Zhao *et al*, 2017). While the gut is postulated to be the “motor” of multiple organ failure (MOF), allowing systemic ingress of gut bacteria and their cellular constituents (Klingensmith & Coopersmith, 2016), the surface pathophysiology involving the intestine may be different between species. Indeed, the surface ratio of the small intestine to the colon is 22‐fold lower in mice than humans (Nguyen *et al*, 2015). The immune system also differs in many ways (Mestas & Hughes, 2004), and the main acute phase proteins produced in sepsis vary between species. Human and murine neutrophils also display numerous differences (Table 1); most striking is the different effect of sepsis on NETosis, which is reduced in humans (Hashiba *et al*, 2015) but enhanced in mice (Meng *et al*, 2012). With respect to platelets, not only do number and size differ between humans and mice, but also their mRNA content (Schmitt *et al*, 2001; Rowley *et al*, 2012).

**Table 1:** Some physiologic and immunologic differences between mice and humans that may affect the host response to infection, the development of sepsis, and its monitoring

	Mice	Humans
Physiology		
Circadian rhythm	Nocturnal	Diurnal
Nutrition	Standardized chow diet	Varied
Glucose levels	↓ after sepsis	↑ after sepsis
Temperature	↓ after sepsis	↑ after sepsis
Metabolic rate	↓ after sepsis	↑ with initial sepsis, normalizes with increasing severity
Immune system		
Predominant white blood cell type	Lymphocyte	Neutrophil
Enzymatic content in neutrophils	Low	High
α‐defensin production by neutrophils	No	Yes
Expression of CXCR1 on neutrophils	No	Yes
NETosis after sepsis	Increased	Decreased
Missing genes	IL‐8, IL‐32, IL‐37, LFA‐3	TLR11, TLR12
	TLR10, Caspase 10	MCP‐5
Main inflammasome player in LPS sensing	Caspase 11	Caspase 4 and 5

A well‐recognized difference between rodents and humans is the very high resistance of rodents to endotoxin and their greater resilience to infection (Warren *et al*, 2010) or sterile insults (Gentile *et al*, 2013). Genes encoding proteins that sense PAMPs and DAMPs, and coding for cytokines and chemokines are not strictly identical (Table 1). The transcriptomic expression of cells within human and murine blood following different severe inflammatory insults poorly correlates (Seok *et al*, 2013), even if an independent re‐analysis of the same dataset yields completely different results (Takao & Miyakawa, 2015).

Another thorny aspect of animal experimentation is that the experimental model of infection strongly influences the host response. Thus, findings from one model may not necessarily be generalizable. For example, the metabolomic signature differed in rats undergoing cecal ligation and puncture (CLP) compared to those receiving an intra‐peritoneal injection of *S. aureus* (Lin *et al*, 2016). Similarly, in two models of intra‐abdominal sepsis (CLP versus administration of a cecal slurry) with similar mortality, addition of tissue ischemia/infarction (cecal ligation) to an infectious process significantly modified the host transcriptomic response (Gentile *et al*, 2014).

Finally in most animal models, the treatment is administered before, concurrent, or soon after the initiation of the infectious insult. By contrast, patients usually develop sepsis over days; often by the time of presentation, organ dysfunction is already well established.

### Inappropriate selection of patients

Agglomerating different types of severe infection under the “sepsis” umbrella has resulted in enrollment of a heterogeneous population of patients into clinical trials. In addition to intrinsic individual heterogeneity reflecting genetic and epigenetic diversity (Fig 2), specific patient characteristics such as underlying comorbidities, obesity, medications, reactivation of asymptomatic viral infections, age, and sex can all modify the overall host response to pathogens, and thus patient outcomes. Individual heterogeneity is impossible to avoid, though selecting patients according to the type of infection would be more feasible. Mechanisms underlying the host response are greatly influenced by the location of the infectious insult. This reflects the fact that immune cells reside within varied molecular and cellular microenvironments in different tissues and, as a consequence, display specific behavior. For example, murine intestinal macrophages are unresponsive to conventional stimuli (Smythies *et al*, 2005); peritoneal macrophages, alveolar macrophages, and blood monocytes are stimulated differently by *Staphylococcus aureus* (Kapetanovic *et al*, 2011); spleen, lung, peritoneal macrophages, and microglial cells express different patterns of transcriptomic and cell surface expression (Gautier *et al*, 2012); and alveolar macrophages fail to develop endotoxin tolerance (Philippart *et al*, 2012). This late phenomenon has also been observed with human alveolar macrophages (Smith *et al*, 1994). Studies in septic patients confirm how different sites of infection affect the systemic response (Gogos *et al*, 2010; Hoser *et al*, 2012). Thus, not surprisingly, specific pathophysiological mechanisms differ between compartments, and therapeutic intervention should be adapted accordingly with patient cohorts more homogenous in terms of the infection site.

Another cause of heterogeneity is the nature of the infectious agent. The PAMPs and toxins released by Gram‐positive and Gram‐negative bacteria are not identical. Most superantigens are derived from Gram‐positive bacteria and induce an unique cytokine profile, including lymphotoxin‐α derived from T‐lymphocytes (Müller‐Alouf *et al*, 1994). This can be observed particularly in patients with *Streptococcal* toxic‐shock syndrome (Sriskandan *et al*, 1996). Endotoxins from Gram‐negative bacteria are highly potent PAMPs inducing the greatest number of gene activations (Schmitz *et al*, 2004). Notably, endotoxin can also be present in patients with Gram‐positive infection due to gut translocation (Opal *et al*, 1999). A strong synergy has been regularly reported between Gram‐positive superantigens and endotoxin or other Toll‐like receptor agonists (Cavaillon, 2018). CD137, a member of the TNF receptor superfamily, is expressed on neutrophils and shown to be protective during Gram‐positive and deleterious during Gram‐negative infections (Nguyen *et al*, 2013).

## Improving the chances of therapeutic success

### Faster and more reliable diagnosis

Significant delays in initiating treatment will impact upon patient outcomes. Highly specific, sensitive, and rapid tests are urgently needed to avoid unnecessary, inappropriate, or ineffective use of antibiotics. Although many host biomarkers have been reported (Parlato & Cavaillon, 2015), none of them (alone or in combination) displays sufficiently high levels of specificity and sensitivity (Parlato *et al*, *2018*). However, various novel approaches show promise in distinguishing sepsis from sterile inflammation using transcriptomics (Sweeney *et al*, 2018a), metabolomics (Mickiewicz *et al*, 2015), or microfluidics (Hassan *et al*, 2017; Ellett *et al*, 2018). Combined analysis of leukocyte biomarkers has also been investigated. Finally, proteomics and transcriptomics have also been proposed to allow discrimination between bacterial and viral infection (Oved *et al*, 2015; Miller *et al*, 2018).

As therapeutic approaches could differ depending on illness severity, signatures stratifying patients according to their risk of death could be of interest. Transcriptomic (Sweeney *et al*, 2018a, 2018b) and cell surface marker (Conway Morris *et al*, 2018) signatures have already been described. More desirably, patients could be identified based on the likelihood of response to a particular treatment. For example, very high serum levels of ferritin could be used as a marker of hemophagocytic lymphohistiocytosis, for which specific treatments such as an IL‐1 receptor antagonist (anakinra) may be suited (Lachmann *et al*, 2018).

Rapid identification of the infectious agent and of its antibiotic sensitivity is also of primary importance. While PCR assays offer good sensitivity and specificity (Salimnia *et al*, 2016), they require time‐consuming blood cultures, and direct identification of bacterial DNA within freshly isolated blood samples is still associated with too many false‐positive and false‐negative results (Fitting *et al*, 2012; Dark *et al*, 2015). A test combining culture‐independent PCR/electrospray ionization‐mass spectrometry technology was launched commercially but, alas, withdrawn as the cost was too prohibitive. However, it displayed 81% sensitivity, 69% specificity, and 97% negative predictive value at 6 hours from sample acquisition (Vincent *et al*, 2015). *Post hoc* analysis of this cohort revealed a higher mortality in patients with molecular test‐positive, culture‐negative samples (O'Dwyer *et al*, 2017).

### Improving translational research

Refinement, reduction, and replacement of animal models should be strongly encouraged. *In vitro* or *ex vivo* studies can be performed directly on human cells and tissues, and these may be complemented by more sophisticated and robust human organoid and organ‐on‐chip models. Experiments using rodent models remain useful and informative; however, their limitations should be humbly recognized. Improvements have been proposed (Osuchowski *et al*, 2018), such as the use of antibiotics, fluids, and clinical isolates instead of laboratory bacterial strains, which may fail to mimic real‐life pathogenesis due to their reduced capacity to make biofilms (Fux *et al*, 2005). The role of wild‐type microbial flora in influencing outcome has only recently been appreciated (Velazquez *et al*, 2019). The wild‐type microbiome acquired by co‐housing specific pathogen‐free mice with their pet‐shop mates allowed the mice to develop a mature immunity capable of generating responses more similar to those of humans (Beura *et al*, 2016). Colonizing experimental mice with a human patient‐derived pathobiome that emerges in critical illness would further allow to better mimic pivotal host–pathobiome interactions (Alverdy & Krezalek, 2017). An appropriate choice of infection model (pneumonia, CLP, or soft tissue infection) with a relevant infection route and pathogen load would also better reflect a given human sepsis type.

Stratification of septic animals according to their predicted probability of survival may help discriminate the effectiveness of novel treatments (Osuchowski *et al*, 2009). Telemetry techniques reveal a variable physiological response of mice subjected to a similar septic insult (Lewis *et al*, 2016); this could be utilized to select only significantly affected individuals for enrollment into a treatment study, to increase homogeneity and improve assessment of the treatment effects. More “real‐life” conditions could be achieved, for instance by the use of aged animals or animals with comorbidities. Two‐hit models, where an initial trauma or infectious episode is followed by induction of a secondary infection, resemble a frequent clinical scenario, and may recapitulate the human host responses better than single hit models (Muenzer *et al*, 2006). Finally, specific aspects of the human immune response could be examined within humanized mice models with added genetic variability from transplantation of stem cells originating from different donors (Skirecki *et al*, 2015; Prince *et al*, 2017).

Non‐rodent models such as rabbits and pigs have some advantages, for example, greater sensitivity to bacterial infection and responses resembling better those observed in humans (Fairbairn *et al*, 2013; Salgado‐Pabon *et al*, 2013; Waterhouse *et al*, 2018). While rhesus monkeys and baboons are highly resistant to endotoxin and have a separate pattern of host response (Barreiro *et al*, 2010), squirrel monkeys have a relatively high sensitivity (Lipton & Fossler, 1974). Studying naturally sick animals (Werners, 2017) or non‐laboratory‐bred animals could also be informative as their immunity and host response will differ.

### Should the main goal be to save life or to improve quality of life after sepsis?

With such a provocative question, we want to address whether trials should aim at improving mortality benefit and/or improving patient selection. With a current hospital mortality rate of 25%, achieving a 20% improvement in mortality with a *P* value below 0.05 requires inclusion of 1,191 patients. Arguments are however made to increase the default *P* value threshold to 0.005 for claims of discovery (Benjamin *et al*, 2018), thus requiring many more patients, or reducing the sample size by a Bayesian approach (Goligher *et al*, 2018). Others argue for adaptive trial designs with simultaneous inclusion of several treatment arms and modifications justified by findings emerging during the trial (Talisa *et al*, 2018). However, adequate group sizes in such a heterogenous critical care population may prove to be problematic.

Another challenge lies in accurate selection of therapeutic targets that have a realistic probability of improving survival from sepsis. Possible ways to improve translational research are discussed below. Furthermore, selection of targets based upon observational clinical trials can also be misleading. There is a confounding effect of illness severity that may not be adequately adjusted for in *post hoc* analyses. For example, a positive fluid balance is linked to worse outcomes, yet sicker patients generally need more fluid replacement and are more likely to have concurrent renal dysfunction. Thus a positive fluid balance may be epiphenomenal rather than necessarily causative of a higher mortality. Furthermore, changes in a blood mediator level linked to a poor outcome can be part of the host response to injury, or even a protective element related to disease tolerance (Medzhitov *et al*, 2012; Bauer *et al*, 2018).

Enhancing survivorship is gaining increasing attention as many of the 70% of septic patients discharged from hospital will suffer from post‐sepsis physical, psychological, and/or cognitive impairments, with consequences for the patients, their family and the healthcare system (Prescott & Angus, 2018). Contributing factors to poor survivorship comprise underlying frailty and comorbidities, degree and duration of illness severity, lack of early rehabilitation, and drugs, such as corticosteroids, sedatives and paralyzing agents. While frailty and underlying comorbidities are not usually correctable, identification of interventions for reversible conditions will hopefully lead to enhanced recovery (Schweickert *et al*, 2009; Wade *et al* 2019).

As described at the beginning of this article, we pointed out that many people die “*with”* rather than “from” sepsis because of their significant underlying comorbidities, which both compromise their initial host response and/or the subsequent recovery pathway. A treatment may be effective in reversing the acute septic illness, yet the patient still dies in the hospital because of frailty and/or preexisting organ dysfunction. Length of stay and duration of mechanical support will also be longer in such patients, confounding any beneficial effect on time to recovery and ICU or hospital discharge in less sick survivors. In view of the many available prognosticators—physiological, biochemical, and molecular—that can identify likely survival at an early stage in the patient's hospital admission, the possibility of better trial stratification is intriguing. Predicted survivors can be separated from predicted non‐survivors. In the former, the focus could be on time to recovery and quality of survivorship with unanticipated death used as a safety signal. On the other hand, a mortality signal can be primarily sought in predicted non‐survivors, with a greater treatment effect being perhaps more likely in this sicker cohort.

### A personalized medicine approach

**Figure 6:**
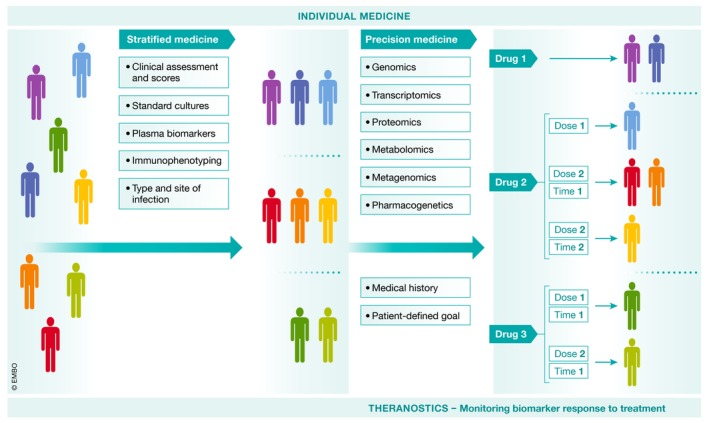
Strategies to enrich treatment‐sensitive subpopulations of patients At the admission, patient is subjected to supportive therapies and samples are taken for laboratory analyses. The flowchart on the left side shows current approach to stratify the patients using relatively simple methods such as clinical scales, mediator concentrations, or activation of immune cells. Then basing on the thresholds patients are qualified or not to a given specific therapy. This approach is already used in some clinical trials and for some biomarkers feasible at the bedside. On the right, a procedure of future individual medicine is presented. It involves a more complicated approach which applies potent analytical platforms to assess the genome, transcriptome, proteinome, and metabolome of the patient. Together with metagenome of the pathogen, the decision is made on the drug to prescribe as well as its dose and timing.

Increasing emphasis is being placed on personalized medicine (Fig 6), and an important aspect to consider is aging. Indeed, the links between immunosenescence and sepsis have been poorly investigated (Martín *et al*, 2017), although immunosenescence is associated with increased sensitivity of aged people to infection (Fulop *et al*, 2018; Mannick *et al*, 2018). However, most experimental sepsis models are performed on murine teenagers. Mortality is far greater in aged mice following CLP (Saito *et al*, 2003) or with polytrauma with pneumonia (Nacionales *et al*, 2015). The elderly animals show enhanced coagulopathy, decreased generation of activated protein C (Starr *et al*, 2015), and a more pronounced cytokine storm.

Blocking or activating specific pathways may be potentially beneficial when a pathway is over‐ or under‐expressed in patients who fare badly, albeit with the caveat that this may represent an adaptive rather than detrimental response. This may apply to the use of IL‐1 receptor antagonism (Shakoory *et al*, 2016), anti‐TNF therapies (Panacek *et al*, 2004), or corticosteroids (König *et al*, 2018), where *post hoc* analyses suggest benefit in those with raised ferritin, raised IL‐6 levels and low IFN‐*γ*/IL10, respectively (Table 2). On the other hand, a *post hoc* analysis of a clinical trial showed that a transcriptomic signature suggestive of a more immunocompetent profile fared significantly worse with steroid therapy (Antcliffe *et al*, 2019).

**Table 2:** Examples of clinical trials that showed benefits in subgroups of septic patients

Drug/intervention	Subgroups	Benefit	Mode of analysis	References
Afelimomab (anti‐tumor necrosis factor F(ab’)2 monoclonal antibody fragment)	IL‐6 > 1,000 pg/ml	28‐day mortality 43.6% vs. 47.6% placebo	Prospective	Panacek *et al* (2004)
GM‐CSF	Monocytic HLA‐DR < 8,000 antibodies per cell	Time of mechanical ventilation 148 ± 103 vs. 207 ± 58 h (placebo), *P* = 0.04	Prospective	Meisel *et al* (2009)
Anakinra (IL‐1 receptor antagonist)	Features of hemophagocytic lymphohistiocytosis (disseminated intravascular coagulation (DIC), thrombocytopenia and hepatobiliary dysfunction)	28‐day mortality 34.6% vs. 64.7% placebo	Re‐analysis of de‐identified data from the phase III randomized interleukin‐1 receptor antagonist trial in severe sepsis	Shakoory *et al* (2016)
Trimodulin (polyclonal immunoglobulin preparation)	CRP ≥ 70 mg/l and IgM ≤ 0.8 g/l	28‐day mortality 11.8% vs. 36.6% placebo (*P* = 0.006)	Exploratory *post hoc*	Welte *et al* (2018)

Analysis of clinical data from more than 20,000 patients identified four distinct clinical phenotypes with different host‐response patterns (Seymour *et al*, 2019). Computer simulations suggested that the distribution of particular phenotypes could affect the trial outcome. Others have suggested identifying patients with a major immunosuppressive profile (Hotchkiss *et al*, 2013; Bermejo‐Martin *et al*, 2016) though this may be less promising than anticipated (Cavaillon *et al*, 2014). A recent study on 700 transcriptomic profiles suggested that patients with bacterial sepsis could be divided into “inflammopathic”, “adaptive”, and “coagulopathic” clusters (Sweeney *et al*, 2018a), with each state potentially corresponding to different therapeutic approaches. A summary of transcriptomic studies exploring subgroup responses can be found in Table 3. While intriguing, such approaches have yet to be tested prospectively, let alone validated.

**Table 3:** Described endotypes of sepsis. Heterogeneity of the host response to sepsis is a major cause of difficulties in the development of effective targeted therapies. By the use of genome‐wide expression assays, different patterns of transcriptomic response (of blood leukocytes) in sepsis are distinguished. Unraveling these patterns creates opportunities to find new pathways that can be targeted in a given subgroup. The term endotype is used to distinguish the transcriptome‐based diversity from the classical phenotypic description

Endotypes	Methodology	Studied group	Implications	References
Subclass A: repression of adaptive immunity and zinc‐related biology Subclass B Subclass C	Genome‐wide expression profiling, unsupervised hierarchical clustering of genes which expression was ≥ 2‐fold changed (comparing to controls) in 25–50% of patients	Children with septic shock (*n* = 98)	Identification of high‐risk subpopulation by subclass An assessment identification of novel therapeutic targets	Wong *et al* (2009)
Subclass A Subclass B	Multiplex mRNA quantification platform to analyze the expression of the 100 subclass‐defining genes	Children with septic shock (*n* = 168)	Development of a method for endotyping pediatric septic shock Identification of endotype (A) associated with the harmful effects of glucocorticosteroids	Wong *et al* (2015)
Mars1: immunosuppression, increase in heme biosynthesis pathway components Mars2: increased expression of genes related to pattern recognition, cytokines, cell growth Mars3: adaptive immunity; IL‐4, NK‐cell signaling Mars4: interferon signaling, pattern recognition, TREM1 signaling	Genome‐wide expression	Sepsis (*n* = 306), validation cohort (*n* = 216), second validation cohort (CAP sepsis *n* = 265)	Mars1 type response is related to poor early‐ and long‐term outcome	Sclicluna *et al* (2017)
SRS1 (Sepsis Response Signature 1): immunosuppression, T‐cell exhaustion, endotoxin tolerance SRS2: proliferation, immune response, cell adhesion	Genome‐wide microchip array, variation in global gene expression by unsupervised hierarchical clustering	Sepsis due to CAP (*n* = 265 and validation cohort *n* = 106)	SRS1 is a predictor of high early mortality	Davenport *et al* (2016)
SRS1: cell death, apoptosis, endotoxin tolerance SRS2: cell adhesion, differentiation, proliferation, immune response	Genome‐wide Microarray, variation in global gene expression	Fecal peritonitis sepsis(*n* = 117) (also comparison with CAP; *n* = 126)	SRS1 is a denominator of high early mortality, but the shift to SRS2 pattern is a marker of favorable prognosis	Burnham *et al* (2017)
Endotype A Endotype B	Retrospective analysis of transcriptomic data using pattern of 100 genes expression	Sepsis (*n* = 549)	Highest mortality in patients < 40 y.o. co‐allocated into endotype A/SRS1. Suggestion of relationship between immunosuppressive response and mortality	Wong *et al* (2017a)
Endotype A Endotype B	Retrospective classification and regression tree analysis of retrospective data to find the smallest discriminatory set of genes	Septic children (*n* = 300); validation group (*n* = 43)	Development of four‐gene based protocol for endotyping of septic children. Potential to identify glucocorticoid responses	Wong *et al* (2017b)
SRS1 SRS2	Genome‐wide microarray, allocation based on the generalized linear model based on 7 genes (from Davenport *et al*, 2016)	Sepsis (*n* = 177)	Hydrocortisone treatment increases mortality in SRS2	Antcliffe *et al* (2019)
Inflammopathic: pro‐inflammatory, complement pathways Adaptive: adaptive immunity and interferon signaling Coagulopathic: platelet degranulation, coagulation cascade	Genome‐wide expression	Retrospective analysis of septic patients (*n* = 700) from 14 trials	Identification of major deregulated pathways in endotypes that can direct selective treatment	Sweeney *et al* (2018a)

CAP, community acquired pneumonia; SRS, sepsis response signature.

Clearly, this branch of diagnostics is still in its relative infancy. Advances in platform technology such as next‐generation sequencing with interrogation of the whole genome will open up important new insights, as will the rapidly advancing areas of metagenomics, metabolomics, and proteomics. The time to access data will be dramatically reduced, enabling results within minutes to hours, a timeline that is crucial for intervention in a critically ill patient. PCR and multiplex protein point‐of‐care technologies already exist and yield results within this timeline; such devices will continue to become more refined and sophisticated. Crucially, standardization, or at least cross‐validation, of the different platforms and computer analytic techniques will be needed to ensure consistency. A further point to consider is the current reliance on blood sampling and extrapolation of results from either predominantly white cells, or circulating humoral mediators (e.g., cytokines, hormones, metabolites), to changes at the organ level. An excellent but sadly overlooked rat CLP study showed markedly different transcriptomic changes between lung, liver, kidney, thymus, spleen, and brain, both spatially and temporally (Chinnaiyan *et al*, 2001). Pathophysiological assumptions made from circulating blood cells may not hold true in the overall scheme.

## Revisiting old strategies or new approaches?

As mentioned earlier, many approaches that were successful in preclinical models and Phase II clinical trials have failed at the larger multi‐centre trial stage. The increasing appreciation of different phenotypes/endotypes within the syndrome of sepsis and *post hoc* analyses of trial data suggesting outcome benefit (e.g., Calfee *et al*, 2018 and statins), or detriment (Antcliffe *et al*, 2019, and steroids) in specific subsets, does suggest that these old agents could be gainfully revisited, especially with the advent of diagnostics enabling rapid subset identification. IL‐1Ra therapy could be considered for patients with a hemophagocytosis profile (Shakoory *et al*, 2016), recombinant soluble thrombomodulin in those with a coagulopathic profile (Kato & Matsuura, 2018), or intravenous immunoglobulin (IVIg) in those with a hyperinflammatory profile (Welte *et al*, 2018). Immunostimulant drugs such as interferon‐gamma and GM‐CSF are also being re‐explored, using lymphopenia as a surrogate for immunosuppression. Non‐drug approaches, such as hemofiltration and blood purification techniques, may also show benefit when targeting specific subsets rather than general populations (e.g., Payen *et al*, 2009; Gaudry *et al*, 2016; Dellinger *et al*, 2018; Hawchar *et al*, 2019).

The same argument should be applied to novel investigational drugs. Many are in a clinical testing phase, but it is imperative that they be tested in optimal population subsets. For example, a dose‐finding phase II trial (Adrenoss‐2) has just been completed for adrecizumab in patients with septic shock and elevated concentrations of circulating bio‐adrenomedullin (Geven *et al*., 2019). Other approaches are also being studied, including electroacupuncture, which showed benefit in endotoxic mice (Torres‐Rosas *et al*, 2014) and more recently, anti‐inflammatory bowel‐protective effects on intestinal functions in patients with sepsis‐induced intestinal dysfunction (Meng *et al*, 2018).

There are multiple other agents being studied at present in preclinical models targeting a wide range of inflammatory, immune, metabolic, bioenergetic, and hormonal pathways (Dalli *et al*, 2014; Tancevski *et al*, 2014; Winkler *et al*, 2017; Rathkey *et al*, 2018; Sham *et al*, 2018). Transfer of mesenchymal stem cells appeared promising in preclinical studies, and phase 1 and 2 clinical trials are ongoing (Keane *et al*, 2017). Preparations of exosomes from mesenchymal stem cells (Chang *et al*, 2018) or endothelial progenitor cells (Zhou *et al*, 2018) also show early promise. How many of these approaches will proceed to clinical trials and then clinical use remains open to question. There may be no magic target to cure sepsis, and a combined approach could be more beneficial than targeting a single molecule, as shown for IL‐1 and IL‐18 in murine sepsis (Vanden Berghe *et al*, 2014), IL‐1 and TNF in a rat model (Russel *et al* 1995), or CD14 and factor XIa in rabbits (Nakamura *et al*, 2017).

## Improving survivorship

While the therapeutic emphasis has long been focused on survival, increasing effort is now being placed on enhancing survivors’ quality of life. Below are a few approaches being examined to improve functionality in different organs.

### Protecting the brain

Minimization or avoidance of iatrogenic factors related to treatment (e.g., prolonged use of benzodiazepine sedation, and adequate pain treatment) and non‐pharmacological modalities (e.g., physiological light cycle, cognitive stimulation, and early mobilization) may prove useful (Souza‐Dantas *et al*, 2016). Existing drugs such as metformin (Tang *et al*, 2017) and minocycline (Adembri *et al*, 2014) show brain protective effects in experimental sepsis. The natural antioxidant berberine improved survival in a rat CLP model but also motor and cognitive functions (Shi *et al*, 2018).

### Improving muscle function

An adequate diet with high protein intake may be pivotal in dealing with critical illness‐induced sarcopenia (Wischmeyer & San‐Millan, 2015), perhaps in conjunction with an individualized physical rehabilitation regimen. Early rehabilitation appears to improve short‐term ICU‐acquired weakness, but not long‐term weakness nor mental status (Fuke *et al*, 2018). Despite contradictory clinical data, neuromuscular electrostimulation reduced muscle atrophy in endotoxemia models (Poulsen *et al*, 2011; Rodriguez *et al*, 2012; Tanaka *et al*, 2016). Use of beta‐blockers to inhibit catabolism was proved to be efficient in burn patients (Herndon *et al*, 2001). Anabolic hormones such as testosterone or growth hormone are also of interest, despite their potential side effects (Rosenthal & Moore, 2015). However, growth hormone increased mortality in critically ill patients (Takala *et al*, 1999).

### Preserving immune functionality

Different strategies are proposed, ranging from boosting reagents (Hotchkiss *et al*, 2013) to agents limiting the inflammatory reaction, such as myeloid suppressor cells (McPeak *et al*, 2017) or regulatory T cells (Heuer *et al*, 2005). Preventing T‐cell exhaustion by anti‐PD‐1/PD‐L1, anti‐CTLA‐4 treatments, or IL‐7 therapy, has also been suggested (Hotchkiss *et al*, 2019a,b; Francois *et al*, 2018). Another approach is to boost immune function by vaccination; a multi‐centre trial using a polyvalent conjugate pneumococcal vaccine is ongoing in the UK (https://clinicaltrials.gov/ct2/show/NCT03565159).

## Conclusions

Tremendous progress in basic science together with numerous clinical/epidemiological studies has increased our knowledge of sepsis. However, these advances have not yet translated into the development of effective new treatments. Rather, they have demonstrated the complexity and heterogeneity of the syndrome, and the need to better target interventions to the right patient subset, at the right time, at an optimal dose and for an optimal duration. This requires better diagnostics and theranostics to first identify suitable patients, and then titrate treatment to an optimal endpoint, rather than adopt a rather naïve and simplistic “one‐size‐fits‐all” philosophy. Such diagnostics and theranostics need to be measured rapidly to enable timely intervention, and suitable targets where modification will impact the outcome need to be identified. These should be tested in improved translational preclinical models that better reflect the human illness with appropriate timing of treatment.

Without such a change in the approach, it is unlikely that the battle against sepsis will succeed. It is also important to recognize that, even with the best care, only a proportion of septic patients can be rescued.

It is also important to rethink treatment goals for survivors as many will suffer from multiple physical, psychological, and cognitive dysfunctions that may be permanent (Tinetti *et al*, 2016). This requires expansion of our understanding of the disease at the molecular and cellular levels. We advocate for clinical studies that have hypotheses that can be carefully tested in relevant long‐term animal models (Efron *et al*, 2015). The bridge between clinical and basic studies in sepsis needs to be rebuilt and made two‐way.

**Pending issues**

(i) Identification of critical pathophysiological events in development of sepsis and distinction between causative, adaptive or bystander phenomena.
(ii) Better understanding of the local tissue‐specific responses during sepsis.
(iii) Improvement of the preclinical models for both basic and translation research: animal models more relevant than rodents (e.g., rabbits, squirrel monkeys, pigs), site of infection frequently found in human sepsis (e.g., lungs…), clinical isolates.
(iv) Development of integrated personalized approach that combines clinical phenotypes, biomarkers, and endotypes unraveled by ‐omics technologies, microfluidic approaches, and artificial intelligence technologies.
(v) Better design of clinical trials to stratify or identify patient populations that may benefit from treatment (prognostic or predictive enrichment) and thus decrease heterogeneity; use of more relevant endpoint (such as quality‐of‐life after sepsis).

## Author contributions

J‐MC, MS, and TS contributed to the conception, the writing, and the editing of the manuscript.

## Conflict of interest

The authors declare that they have no conflict of interest.

## For more information

(i) https://www.sccm.org/SurvivingSepsisCampaign/Home
(ii) https://www.worldsepsisday.org/
(iii) http://internationalsepsisforum.com
(iv) http://www.egis-online.eu/
